# Supplementary material for: Belief updating in bipolar disorder predicts time of recurrence
Source: eLife. 2020 Nov 10;9:e58891. doi: 10.7554/eLife.58891 (PMC7655098; doi:10.7554/eLife.58891)
Supplement: Supplementary file 1. [file elife-58891-supp1.doc]

**Supplementary File 1**

Sample descriptive and task-related variables

**Supplementary File 1a: Descriptive patients**

| at baseline |  | |
| --- | --- | --- |
| Sex (Male) | | 19 (52.8%) |
| Age | | 44.08 (12.6) |
| Education (years) | | 12.91 (3.5) |
| Duration illness (years) | | 12.22 (8.64) |
| Number Previous Episodes | | 7.69 (6.65) |
| History of Psychotic symptoms | | 29 (80.6%) |
| Bipolar II | | 4 (11.1%) |
| BDI-II | | 7.61 (7.05) |
| LOT-R | | 14.47 (4.59) |
| Antipsychotic | | 17 (47.2%) |
| Mood Stabilizer | | 30 (83.3%) |
| *Lithium* | | 21 (58.3%) |
| Antidepressant | | 7 (19.4%) |
| At follow-up (5 years) | |  |
| Time in euthymia (months) | | 17.99 (20.22) |
| (min-max) | | [1-60] |
| Relapsed | | 30 (83.3%) |
| Next Episode Polarity (manic) | | 17 (56.7%) |

**Note.** Values represent mean (SD) unless stated otherwise. In squared bracket the min-max values. LOTR=Life Orientation Test-Revised.

**Supplementary File 1b: Task related variables**

| **Task scores** | |  |
| --- | --- | --- |
| Update bias | | 3.08 (1.94) |
| **Subjective Scales Questionnaire** | |  |
| Familiar | | .38 (.11) ^v^ |
| Prior Experience | | .32 (.09) ^v^ |
| Vividness | | .51 (.13) ^v^ |
| Emotional Arousal | | .42 (.11) ^v^ |
| Negativity | | .25 (.11) ^v^ |
| **Task-related variables** |  | |
| Mean first estimate | | 23.07 (1.86) ^v^ |
| Estimation Error | | .69 (.53) |
| Memory Errors | | -.99 (1.51) |
| Number of trials | | -.86 (2.34) |
| RT at first estimate (msec) | | -6.92 (81.49) |
| RT at second estimate (msec) | | -52.95 (62.03) |

**Note.** ^v^ Effect of valence; p<0.05 tested using a one-sample t test on the bias scores (difference between good and bad news). Values represent mean bias (SE) calculated as the difference between good news and bad news. Subjective scale questionnaires and memory errors were available only for n=35 at follow-up. RT, reaction time;
