# Supplementary material for: Belief updating in bipolar disorder predicts time of recurrence
Source: eLife. 2020 Nov 10;9:e58891. doi: 10.7554/eLife.58891 (PMC7655098; doi:10.7554/eLife.58891)
Supplement: Supplementary file 2. [file elife-58891-supp2.doc]

**Supplementary File 2**

Regression coefficients for Model 1 when the predictor of interest are the update bias, update from good news and update from bad news, and for Model 2

**Supplementary File 2a**: Regression Coefficients of Model 1 with Update Bias as a predictor of interest

|  | **beta** | **t** | **p** |
| --- | --- | --- | --- |
| Constant |  | 4.257 | <.001 |
| Update Bias | .589 | 3.264 | .004 |
| mean first estimate | -2.743 | -3.331 | .003 |
| Δ number of trials | 2.015 | 3.275 | .003 |
| Δ estimation error | .760 | 1.825 | .082 |
| Δ RT first estimate | -.085 | -.417 | .681 |
| Δ RT second estimate | .246 | 1.596 | .125 |
| Δ memory errors | .092 | .551 | .587 |
| Δ vividness | -.191 | -.732 | .472 |
| Δ familiarity | .119 | .440 | .664 |
| Δ prior experience | -.419 | -2.006 | .057 |
| Δ emotional arousal | .633 | 2.516 | .020 |
| Δ negativity | -.361 | -1.729 | .098 |

**Note**. Dependent Variable: Future Time in euthymia log transformed. Δ (delta) refers to the difference in each parameter between good and bad news trials. R^2^ = 0.630; p = 0.010; Constant: B = 11.034, 95%CI = 5.66 - 16.41.

**Supplementary File 2b**: Regression Coefficients of Model 1 with Update from Good News as a predictor of interest

|  | **beta** | **t** | **p** |
| --- | --- | --- | --- |
| Constant |  | 3.732 | <.001 |
| Update from Good News | .570 | 3.467 | .002 |
| mean first estimate | -2.225 | -2.972 | .007 |
| Δ number of trials | 1.714 | 3.031 | .006 |
| Δ estimation error | .435 | 1.140 | .266 |
| Δ RT first estimate | -.012 | -.059 | .954 |
| Δ RT second estimate | .233 | 1.537 | .139 |
| Δ memory errors | .038 | .247 | .807 |
| Δ vividness | .019 | .073 | .943 |
| Δ familiarity | .115 | .434 | .669 |
| Δ prior experience | -.379 | -1.896 | .071 |
| Δ emotional arousal | .540 | 2.202 | .038 |
| Δ negativity | -.287 | -1.391 | .178 |

**Note**. Dependent Variable: Future Time in euthymia log transformed. Δ (delta) refers to the difference in each parameter between good and bad news trials. R^2^ = 0.630; p = 0.010; Constant: B = 8.66, 95%CI = 3.85 – 13.47.

**Supplementary File 2c**: Regression Coefficients of Model 1 with Update from Bad News as a predictor of interest

|  | **beta** | **t** | **p** |
| --- | --- | --- | --- |
| Constant |  | 2.953 | .007 |
| Update from Bad News | -.276 | -1.265 | .219 |
| mean first estimate | -2.102 | -2.164 | .042 |
| Δ number of trials | 1.483 | 2.088 | .049 |
| Δ estimation error | .529 | 1.043 | .308 |
| Δ RT first estimate | -.175 | -.733 | .471 |
| Δ RT second estimate | .299 | 1.661 | .111 |
| Δ memory errors | -.067 | -.359 | .723 |
| Δ vividness | -.337 | -1.074 | .294 |
| Δ familiarity | .116 | .363 | .720 |
| Δ prior experience | -.257 | -1.079 | .292 |
| Δ emotional arousal | .613 | 2.049 | .053 |
| Δ negativity | -.419 | -1.698 | .104 |

**Note**. Dependent Variable: Future Time in euthymia log transformed. Δ (delta) refers to the difference in each parameter between good and bad news trials. R^2^ = 0.488; p = 0.124; Constant: B = 9.46, 95%CI = 2.82 - 16.1.

**Supplementary File 2d:** Regression Coefficients of Model 2 controlling for other clinical variables

|  | **beta** | **t** | **p** |
| --- | --- | --- | --- |
| Constant |  | 3.197 | .009 |
| Update Bias | .741 | 4.421 | .001 |
| mean first estimate | -2.334 | -3.093 | .010 |
| Δ number of trials | 1.691 | 3.027 | .012 |
| Δ estimation error | .735 | 1.499 | .162 |
| Δ RT first estimate | .012 | .045 | .965 |
| Δ RT second estimate | .445 | 2.920 | .014 |
| Δ memory errors | .144 | .820 | .430 |
| Δ vividness | -.015 | -.060 | .953 |
| Δ familiarity | .052 | .133 | .897 |
| Δ prior experience | -.337 | -1.431 | .180 |
| Δ emotional arousal | .404 | 1.787 | .101 |
| Δ negativity | -.249 | -1.326 | .212 |
| Gender (male) | .101 | .627 | .544 |
| Age | -.040 | -.182 | .859 |
| Years of education | .125 | .956 | .359 |
| Bipolar (type I) | .288 | 1.782 | .102 |
| History of psychotic symptoms | -.114 | -.382 | .710 |
| Mood stabilizers | -.208 | -.930 | .373 |
| Lithium | .383 | 1.744 | .109 |
| Antidepressants | -.429 | -2.329 | .040 |
| Antipsychotics | -.343 | -1.554 | .149 |
| Depressive symptoms at BDI-II | -.015 | -.082 | .936 |
| Duration of Illness (years) | .072 | .426 | .678 |

**Note**. Dependent Variable: Future Time in euthymia log transformed. Δ (delta) refers to the difference in each parameter between good and bad news trials. R^2^ = 0.893; p = 0.011; Constant: B = 8.825, 95%CI = 2.75 – 14.9.
