# Supplementary material for: Belief updating in bipolar disorder predicts time of recurrence
Source: eLife. 2020 Nov 10;9:e58891. doi: 10.7554/eLife.58891 (PMC7655098; doi:10.7554/eLife.58891)
Supplement: Supplementary file 3. [file elife-58891-supp3.docx]

**Supplementary File 3**

**Correlations among the independent variables included in the Models**

| **2** | -.106 |  |  |  |  |  |  |  |  |  |  |  |  |  |  |  |  |  |  |  |  |  |  |  |  |
| --- | --- | --- | --- | --- | --- | --- | --- | --- | --- | --- | --- | --- | --- | --- | --- | --- | --- | --- | --- | --- | --- | --- | --- | --- | --- |
|  | (.537) |  |  |  |  |  |  |  |  |  |  |  |  |  |  |  |  |  |  |  |  |  |  |  |  |
| **3** | -.205 | .918 |  |  |  |  |  |  |  |  |  |  |  |  |  |  |  |  |  |  |  |  |  |  |  |
|  | (.23) | (<.001) |  |  |  |  |  |  |  |  |  |  |  |  |  |  |  |  |  |  |  |  |  |  |  |
| **4** | -.118 | .769 | .521 |  |  |  |  |  |  |  |  |  |  |  |  |  |  |  |  |  |  |  |  |  |  |
|  | (.491) | (<.001) | (.001) |  |  |  |  |  |  |  |  |  |  |  |  |  |  |  |  |  |  |  |  |  |  |
| **5** | .063 | -.31 | -.134 | -.57 |  |  |  |  |  |  |  |  |  |  |  |  |  |  |  |  |  |  |  |  |  |
|  | (.717) | (.066) | (.434) | (<.001) |  |  |  |  |  |  |  |  |  |  |  |  |  |  |  |  |  |  |  |  |  |
| **6** | .123 | .156 | .192 | .064 | .057 |  |  |  |  |  |  |  |  |  |  |  |  |  |  |  |  |  |  |  |  |
|  | (.475) | (.362) | (.263) | (.709) | (.74) |  |  |  |  |  |  |  |  |  |  |  |  |  |  |  |  |  |  |  |  |
| **7** | -.462 | .201 | .211 | .266 | -.226 | -.02 |  |  |  |  |  |  |  |  |  |  |  |  |  |  |  |  |  |  |  |
|  | (.005) | (.247) | (.223) | (.123) | (.192) | (.909) |  |  |  |  |  |  |  |  |  |  |  |  |  |  |  |  |  |  |  |
| **8** | -.005 | -.2 | -.351 | .155 | -.058 | -.045 | .044 |  |  |  |  |  |  |  |  |  |  |  |  |  |  |  |  |  |  |
|  | (.975) | (.25) | (.039) | (.374) | (.742) | (.796) | (.804) |  |  |  |  |  |  |  |  |  |  |  |  |  |  |  |  |  |  |
| **9** | .089 | .073 | -.057 | .252 | .209 | .306 | .079 | .655 |  |  |  |  |  |  |  |  |  |  |  |  |  |  |  |  |  |
|  | (.612) | (.678) | (.744) | (.145) | (.228) | (.074) | (.65) | (<.001) |  |  |  |  |  |  |  |  |  |  |  |  |  |  |  |  |  |
| **10** | .13 | -.092 | -.166 | .156 | .086 | .105 | .231 | .563 | .662 |  |  |  |  |  |  |  |  |  |  |  |  |  |  |  |  |
|  | (.456) | (.599) | (.34) | (.371) | (.623) | (.55) | (.181) | (<.001) | (<.001) |  |  |  |  |  |  |  |  |  |  |  |  |  |  |  |  |
| **11** | -.168 | -.07 | -.124 | .045 | .13 | -.031 | .062 | .637 | .498 | .296 |  |  |  |  |  |  |  |  |  |  |  |  |  |  |  |
|  | (.333) | (.688) | (.478) | (.796) | (.458) | (.861) | (.721) | (<.001) | (.002) | (.084) |  |  |  |  |  |  |  |  |  |  |  |  |  |  |  |
| **12** | -.055 | -.074 | -.138 | -.04 | .213 | -.075 | -.052 | .344 | .358 | .13 | .725 |  |  |  |  |  |  |  |  |  |  |  |  |  |  |
|  | (.753) | (.673) | (.428) | (.818) | (.219) | (.67) | (.768) | (.043) | (.035) | (.458) | (<.001) |  |  |  |  |  |  |  |  |  |  |  |  |  |  |
| **13** | -.047 | -.367 | -.367 | -.166 | .241 | -.227 | -.202 | .396 | .194 | .449 | .233 | -.012 |  |  |  |  |  |  |  |  |  |  |  |  |  |
|  | (.785) | (.028) | (.028) | (.334) | (.157) | (.182) | (.244) | (.018) | (.263) | (.007) | (.179) | (.944) |  |  |  |  |  |  |  |  |  |  |  |  |  |
| **14** | .032 | .226 | .236 | .115 | -.065 | -.093 | -.089 | -.051 | -.077 | -.182 | -.051 | .016 | -.312 |  |  |  |  |  |  |  |  |  |  |  |  |
|  | (.853) | (.185) | (.167) | (.503) | (.704) | (.591) | (.61) | (.77) | (.658) | (.295) | (.77) | (.927) | (.064) |  |  |  |  |  |  |  |  |  |  |  |  |
| **15** | .151 | .175 | .168 | -.03 | .089 | .187 | -.134 | .022 | .268 | .158 | -.042 | -.025 | .073 | .158 |  |  |  |  |  |  |  |  |  |  |  |
|  | (.379) | (.309) | (.327) | (.861) | (.607) | (.275) | (.441) | (.902) | (.12) | (.366) | (.811) | (.888) | (.672) | (.356) |  |  |  |  |  |  |  |  |  |  |  |
| **16** | -.015 | -.152 | -.116 | .016 | -.075 | .054 | -.101 | .154 | -.053 | -.124 | .24 | .031 | .234 | -.161 | -.221 |  |  |  |  |  |  |  |  |  |  |
|  | (.933) | (.376) | (.502) | (.928) | (.664) | (.753) | (.566) | (.376) | (.763) | (.479) | (.166) | (.86) | (.169) | (.348) | (.196) |  |  |  |  |  |  |  |  |  |  |
| **17** | -.107 | .052 | .06 | .076 | .108 | .206 | -.167 | .145 | .14 | -.092 | .271 | .081 | .26 | -.162 | -.008 | .788 |  |  |  |  |  |  |  |  |  |
|  | (.533) | (.765) | (.73) | (.661) | (.529) | (.228) | (.338) | (.407) | (.422) | (.599) | (.115) | (.645) | (.125) | (.346) | (.963) | (<.001) |  |  |  |  |  |  |  |  |  |
| **18** | -.119 | -.139 | -.108 | -.056 | .09 | .082 | .236 | -.012 | .053 | .061 | -.045 | .105 | -.159 | -.019 | -.434 | -.248 | -.245 |  |  |  |  |  |  |  |  |
|  | (.491) | (.418) | (.529) | (.747) | (.60) | (.636) | (.172) | (.947) | (.763) | (.726) | (.796) | (.548) | (.355) | (.913) | (.008) | (.144) | (.15) |  |  |  |  |  |  |  |  |
| **19** | 1.34 | -1.69 | -2.22 | -.68 | .51 | .23 | -1.62 | 1.48 | .30 | .00 | .16 | -.41 | .51 | -.04 | 1.41 | -.17 | .98 | .07 |  |  |  |  |  |  |  |
|  | (.19) | (.10) | (.03) | (.50) | (.61) | (.82) | (.12) | (.15) | (.77) | (.99) | (.87) | (.69) | (.62) | (.97) | (.17) | (.86) | (.33) | (.94) |  |  |  |  |  |  |  |
| **20** | 1.29 | .86 | .77 | .43 | -.53 | 1.56 | -1.34 | -.71 | -.36 | -.43 | .42 | .88 | -.86 | 1.01 | -.49 | .68 | 1.67 | .44 | .892 |  |  |  |  |  |  |
|  | (.21) | (.40) | (.45) | (.67) | (.60) | (.13 | (.19) | (.48) | (.72) | (.67) | (.68) | (.38) | (.40) | (.32) | (.63) | (.50) | (.10) | (.66) | (.345) |  |  |  |  |  |  |
| **21** | -0.97 | -1.63 | -1.96 | .04 | 1.42 | -1.73 | .74 | 1.60 | 1.60 | 3.43 | 1.10 | .72 | 3.92 | -.05 | .64 | -.51 | -.84 | -.12 | 1.21 | 2.68 |  |  |  |  |  |
|  | (.34) | (.11) | (.06) | (.97) | (.17) | (.09) | (.46) | (.12) | (.12) | (<.001) | (.28) | (.48) | (<.001) | (.96) | (.53) | (.61) | (.41) | (.91) | (.271) | (.101) |  |  |  |  |  |
| **22** | 0.48 | -1.02 | -.95 | -1.02 | .25 | -1.11 | -1.64 | -.26 | -.08 | .80 | -.25 | .77 | 1.34 | .06 | 1.24 | -1.84 | -1.53 | -.76 | .022 | .225 | 4.29 |  |  |  |  |
|  | (.64) | (.32) | (.35) | (.32) | (.81) | (.28) | (.11) | (.80) | (.94) | (.43) | (.81) | (.45) | (.19) | (.95) | (.23) | (.07) | (.14) | (.45) | (.881) | (.635) | (.038) |  |  |  |  |
| **23** | 0.47 | .02 | -.67 | 1.41 | -1.80 | -1.54 | .73 | 1.42 | .49 | 1.79 | -.12 | .29 | .76 | .41 | 1.05 | -1.18 | -.90 | -1,11 | .538 | 6.3 | 6.93 | 10.0 |  |  |  |
|  | (.64) | (.98) | (.51) | (.17) | (.08) | (.13) | (.47) | (.16) | (.63) | (.08) | (.91) | (.77) | (.45) | (.69) | (.30) | (.25) | (.38) | (.27) | (.463) | (.012) | (.008) | <.001 |  |  |  |
| **24** | -0.08 | -.27 | -.36 | -.06 | -2.02 | -1.23 | -.69 | -1.30 | -4.21 | -2.16 | -1.32 | -1.26 | -.24 | -.43 | -1.44 | -.37 | -1.39 | .48 | .343 | .089 | 3.04 | .035 | .005 |  |  |
|  | (.94) | (.79) | (.73) | (.95) | (.05) | (.23) | (.49) | (.20) | (<.001) | (.04) | (.20) | (.22) | (.81) | (.67) | (.16) | (.72) | (.18) | (.63) | (.558) | (.766) | .081 | (.851) | (.943) |  |  |
| **25** | -1.29 | .52 | .72 | .44 | -.21 | -.68 | 1.69 | 1.45 | 1.80 | 1.06 | 2.66 | 1.62 | .12 | .53 | -.69 | 1.20 | -.31 | .65 | 4.1 | .014 | .066 | 3.77 | .385 | .066 | 1 |
|  | (.21) | (.60) | (.48) | (.66) | (.84) | (.50) | (.10) | (.16) | (.08) | (.30) | (.01) | (.12) | (.91) | (.60) | (.50) | (.24) | (.76) | (.52) | (.043) | (.906) | (.797) | (.052) | (.535) | (.797) | - |
|  | **1** | **2** | **3** | **4** | **5** | **6** | **7** | **8** | **9** | **10** | **11** | **12** | **13** | **14** | **15** | **16** | **17** | **18** | **19** | **20** | **21** | **22** | **23** | **24** | **25** |
|  | **UB** | **MFE** | **#** | **EE** | **RT1** | **RT2** | **MEM** | **VIV** | **FAM** | **PRIOR** | **EMO** | **NEG** | **AGE** | **EDU** | **BDI** | **DUR** | **EPIS** | **LOTR** | **GEN** | **BIP** | **PSY** | **MS** | **LIT** | **AD** | **AP** |

**Note**. DF=36 if not otherwise specified. Test reported are the Pearson coefficients between continuous variables (1-18); Chi-squared between binary variables (19-25) and t-test when looking the association between continuous and binary variables. In grey significant association (p<.05). In parenthesis the p-values. UB=Update Bias; MFE=Mean First Estimate; #=Delta Number events; EE=Delta Estimation Error; RT1= Delta Reaction Times Session 1; RT2=Delta Reaction Times Session 2; MEM= Delta Memory Errors (DF=35); VIV= Delta Vividness (DF=35); FAM=Delta Familiarity (DF=35); PRIOR=Delta Prior experience (DF=35); EMO=Delta Emotional arousal (DF=35); NEG=Delta Negativity (DF=35); EDU=Years of Education; BDI=Depressive symptoms at BDI-II; DUR=Duration of Illness in years; EPIS=Number of previous Episodes; LOTR= Trait optimism at LOT-R; GEN= gender (coded as 0=male and 1=female); BIP= bipolar type (coded as 1=type I and 2=type II); PSY=history of psychotic symptoms, MS=mood stabilizers, LI= lithium, AD=antidepressants and AP=antipsychotics (all coded as 0=no, 1=yes).
